# A search algorithm for identifying likely users and non-users of marijuana from the free text of the electronic medical record

**DOI:** 10.1371/journal.pone.0193706

**Published:** 2018-03-06

**Authors:** Salomeh Keyhani, Marzieh Vali, Beth Cohen, Alexandra Woodbridge, Melanie Arenson, Elnaz Eilkhani, Christina Aivadyan, Deborah Hasin

**Affiliations:** 1 San Francisco VA Medical Center, San Francisco, CA, United States of America; 2 University of California San Francisco, Department of Medicine, San Francisco, CA, United States of America; 3 Tulane University School of Medicine, New Orleans, Louisiana, United States of America; 4 University of Maryland, Department of Psychology, College Park, Maryland, United States of America; 5 New York State Psychiatric Institute, New York, NY, United States of America; 6 Department of Epidemiology, Mailman School of Public Health, Columbia University, New York, NY, United States of America; Centre for Addiction and Mental Health, CANADA

## Abstract

**Background:**

The harmful effects of marijuana on health and in particular cardiovascular health are understudied. To develop such knowledge, an efficient method of developing an informative cohort of marijuana users and non-users is needed.

**Methods:**

We identified patients with a diagnosis of coronary artery disease using ICD-9 codes who were seen in the San Francisco VA in 2015. We imported these patients’ medical record notes into an informatics platform that facilitated text searches. We categorized patients into those with evidence of marijuana use in the past 12 months and patients with no such evidence, using the following text strings: “marijuana”, “mjx”, and “cannabis”. We randomly selected 51 users and 51 non-users based on this *preliminary* classification, and sent a recruitment letter to 97 of these patients who had contact information available. Patients were interviewed on marijuana use and domains related to cardiovascular health. Data on marijuana use collected from the medical record was compared to data collected as part of the interview.

**Results:**

The interview completion rate was 71%. *A*mong the 35 patients identified by text strings as having used marijuana in the previous year, 15 had used marijuana in the past 30 days (positive predictive value = 42.9%). The probability of use in the past month increased from 8.8% to 42.9% in people who have these keywords in their medical record compared to those who did not have these terms in their medical record.

**Conclusion:**

Methods that combine text search strategies for participant recruitment with health interviews provide an efficient approach to developing prospective cohorts that can be used to study the health effects of marijuana.

## Introduction

Marijuana use for medical purposes is now legal in 26 states and in Washington DC, and in addition, is now legal for recreational use in multiple states [1–3]. Furthermore, over the last several years, marijuana use has increased among the US adult population [4–8]. Although numerous psychosocial and cognitive consequences are associated with marijuana use, Americans increasingly perceive marijuana use as safe and as offering health benefits for some conditions [2,9]. Despite rising use and perception of decreased risk compared to alcohol and tobacco, the potentially harmful physical effects of marijuana have been inadequately studied.

Smoking tobacco is well known to cause numerous health problems, e.g., chronic lung disease, cancer, and cardiovascular disease. Compared to tobacco smoke, marijuana smoke has higher concentrations of particulate matter, toxins, and tar levels. Therefore, chronic use could plausibly lead to similar health problems [10–13]. An area of particular concern is the impact of marijuana use on cardiovascular health, the main cause of morbidity and mortality in the US [14].

While understanding the relationship between marijuana use and cardiovascular outcomes is important, a challenge in beginning to understand these relationships lies in developing prospective cohorts with sufficient marijuana exposure to facilitate research. Multiple studies on the effect of marijuana use on various domains of health have reported limitations in the literature due to small sample sizes with insufficient exposure [15–17]. We therefore developed and tested a method to capture this information from the free text of notes stored in VA electronic medical records.

The method we developed and tested consists of using string searches of medical notes to develop a prospective cohort of older veterans who differ on their level of marijuana exposure. In this study, we demonstrate the feasibility of this method to efficiently develop a prospective cohort of older veterans using text search methods.

## Methods

### Development phase

We first developed a lexicon describing marijuana mentions in the text of medical record notes. Through an iterative process we searched through the text notes of patients in the Veterans Health Administration (VA) to identify how the marijuana use was described. The terms "marijuana", "cannabis","mjx" and “mj” were identified as potential search terms. Review of notes with corresponding terms demonstrated that “marijuana”, “cannabis”, and “mjx”, were the terms most frequently used to describe marijuana use in VA progress notes. “MJ” was discarded because of overlap with abbreviations for temporomandibular joint (TMJ). Before we built a more sophisticated natural language tool, we examined whether identifying marijuana, cannabis and mjx “mentions” in patient notes were sufficient to identify current or former users of marijuana. We used this approach for two main reasons. First, we determined that when clinicians mention a *specific psychoactive substance* (e.g., marijuana or cocaine) in medical progress notes, the mention suggests current or former use and not lack of use. In other words, the presence of a word denoting marijuana use may be sufficient to preliminarily identify users. Second, the VA Informatics and Computing Infrastructure provides a search function that facilitates searching clinical notes for specific word strings. In this study, we examined whether we could use this search function to identify a cohort of marijuana users with sufficient exposure to examine the cardiovascular health risks of marijuana.

### Implementation phase

Using data from an existing cohort of hospitalized Veterans, we first identified patients who were 65 to 67 years old and had a diagnosis of coronary artery disease using ICD-9 codes. We focused on this group because studying the cardiovascular effects of marijuana in a younger, healthier prospective cohort would require substantially longer follow-up. We then limited this sample to patients who had one primary care visit at the San Francisco Veterans Administration (VA) in 2015 to ensure the most recent data on marijuana use was available. The San Francisco VA serves a large geographic region in Northern California extending from San Francisco to small towns in rural areas, and thus cares for a diverse population. We identified 210 patients in this cohort with coronary artery disease who were 65 to 67 years old and who received care in 2015. We categorized these patients into 62 patients with evidence of marijuana use documented in the past 12 months and 148 patients with no evidence of marijuana use using the following text strings: “marijuana”, “mjx”, and “cannabis”. We randomly selected 51 users and 51 non-users based on this *preliminary* classification of marijuana use. Three subjects were deceased, leaving 50 potential users and 49 potential non-users for a total of 99 patients.

### Validation phase

Among the 99 patients, 2 did not have phone numbers and clear contact information available in their medical record. The remaining 97 patients were sent a letter that described the study consisting of a “cardiovascular lifestyle interview” focused on understanding the relationship between cardiovascular events and lifestyle factors such as physical activity, mood, sleep, use of tobacco, and drugs. Standardized and validated instruments were used to assess marijuana use and amount of use (joint-years) (CARDIA and NESARC)[18,19], tobacco use (PRISM)[20], second-hand tobacco exposure (National Health Interview Survey Tobacco Questions)[21], physical activity (Godin Leisure-Time Exercise Questionnaire)[22], alcohol use (AUDIT-C)[23], substance abuse (CARDIA)[24], depression (PHQ-9)[25,26], post-traumatic stress disorder (Primary Care PTSD screen)[27], self-reported health (SF-36)[28], and socioeconomic status (health and retirement survey)[29]. The letter informed potential participants that they would receive a follow-up phone call unless they called the study contact telephone number and left a message saying that they did not wish to participate in the study.

When potential participants were called, a verbal script was used that had been developed and customized for different anticipated scenarios. Specifically, participants were asked if they received a letter describing the study and whether they had read it. If they had not read it, the letter was read to the participant. Patients were then asked if they had any questions about the study, informed that they would receive a $20 gift card for participation, and if they agreed to participation, consented over the phone. The UCSF Human Research Protection Program approved this research and provided a waiver of written consent and a HIPAA waiver.

### Analysis

We report simple descriptive statistics. We estimated the past year of marijuana in the form of joint-years. One joint-year is equivalent to one joint per day for 365 days.

## Results

### Study recruitment rate

Among the 97 patients called, 1 patient called and left a message that they do not want to participate, 20 patients declined to participate during the phone call, and 7 could not be reached after an average of 8 telephone calls. A total of 69 patients completed the interview, leading to a *recruitment rate of 71%*. The average time to administer the health interview was 21 minutes and ranged from 13 minutes to 50 minutes.

### Concordance of text search with patient self report

**Fig 1:**
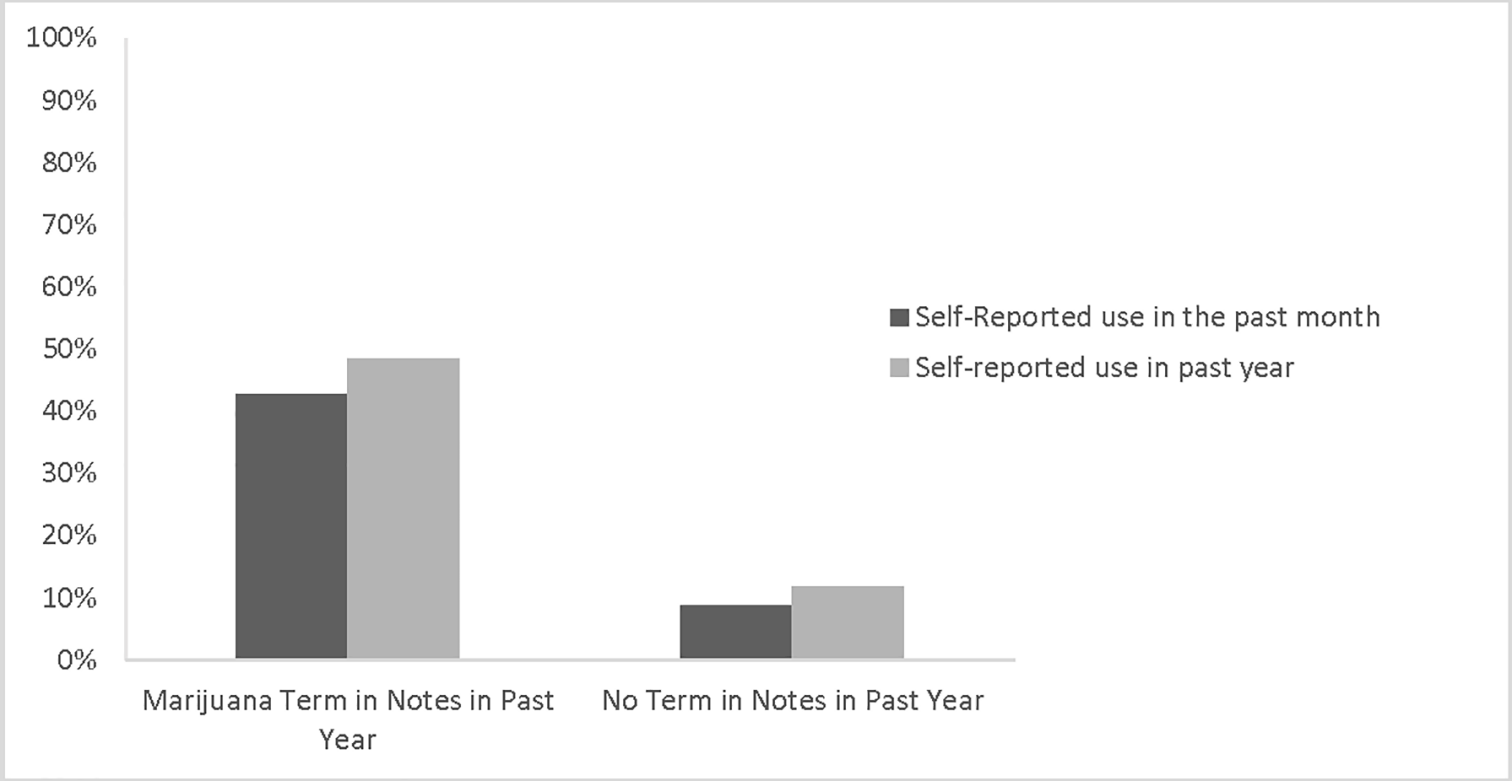
Self reported marijuana use categorized by marijuana use in the chart.

Among the 35 patients identified by text mining as having a marijuana term in their notes in the previous year, 15 had used marijuana in the past 30 days (positive predictive value = 42.9%), 17 self-reported using marijuana in the past year (positive predictive value = 48.6%) and 33 had used marijuana in their lifetime (positive predictive value = 94.3%). Among those not identified by text mining as having a marijuana term in their notes, 3 had used marijuana in the past 30 days. Lifetime ever use also differed based on these terms, with 94.3% of the patients who had a marijuana term in their notes reported ever use and 67.6% of those without a term reported ever use (p = .0016). In other words, the probability of use in the past month increased from 8.8% to 42.9% in people who have these keywords in their medical record compared to those who did not have these terms. The probability of life time ever use also increased from 67.6% to 94.3% among those with these terms in their chart compared to participants without these terms in their chart (Table 1, Fig 1).

**Table 1:** Concordance between text search method and patient interview.

	Marijuana term in notes in past year (n = 35)	No term in notes in past year (N = 34)
Self-Reported use in the past month	15 (42.9%)	3 (8.8%)
Self-Reported use in past year	17 (48.6%)	4 (11.8%)
Self-Report Non-Use	2 (5.7%)	11 (32.4%)
Ever use	33 (94.3%)	23 (67.6%)

P <0.01

### Gradations of current marijuana use based on self-report

Among the 18 patients who reported current use (use in past 30 days), 16 predominantly smoked marijuana (88%), and the remaining two exclusively used other forms of marijuana. Among users, 38.8% smoked marijuana daily, 11.1% smoked at least once a week but not daily, and 5.5% smoked 2 to 3 times per month. Current smokers on average smoked 0.75 joint-years in the past year. Ten of the smokers also used other forms of marijuana (e.g. vaping, topical agents and edibles).

### Other selected patient characteristics

The differences between the two groups were not statistically significant given the small sample size (Table 2), but they strongly suggest baseline differences between current users and non-current users. For example, marijuana users were more likely to smoke tobacco (33.3% vs. 17.6%), drink more than 6 drinks on any occasions (27.8% vs. 17.6%), and have lower rates of physical activity (8.26 vs. 12.65 mean weekly exercise metabolic equivalents).

**Table 2:** Baseline characteristics of cohort.

	Current marijuana users (n = 18)*	Non- current users (n = 51)
Overall health good/very good	44.4%	47.1%
Mean weekly exercise (METs)	8.28	12.65
>6 drinks on one occasion	27.8%	17.6%
Tobacco Abuse		
Current	33.3%	17.6%
Former	50.0%	58.8%
Never	16.7%	23.5%
Positive Depression Screen	33.3%	23.5%
Positive PTSD Screen	55.6%	39.2%
Current illicit drug use**	0%	2.0%

*Current use: use of marijuana in the past 30 days

** e.g., cocaine, amphetamines

## Discussion

An Institute of Medicine report published in 1999 cautioned that marijuana use may present a serious problem for older subjects, particularly those with cardiovascular disease [30]. However, this relationship has never been examined in a prospective cohort study. Identifying a large cohort of current marijuana users with sufficient current marijuana exposure through standard research screening methods such as a mail, telephone, or web based screening is costly and challenging. In this study, we demonstrated that within a large health system, an automated string search of medical record notes in combination with standard survey methods can be used to efficiently develop a prospective cohort of current users and non-users.

Our proposed method for cohort construction leverages information available in the free text of the medical record for rapid prospective cohort construction. The hybrid approach that combines a telephone health interview with data collected as part of routine care improves feasibility of a first assessment of the effect of marijuana use on health. The data collected through the health interview further validates the proposed method for cohort construction and is in line with the health characteristics of marijuana users collected in other studies.[18, 24] Our proposed approach reduces the resources required to conduct a prospective cohort study and demonstrates a feasible and efficient study recruitment method. These methods can potentially be used to develop other cohorts using data from other large health care systems.

Multiple study limitations are noted. We tested our recruitment approach in only one facility. However, to determine if our text search methods were generalizable to the VA system, we used the same methods to search one year of text notes of patients in 2015 in a cohort of hospitalized VA patients from other states where marijuana is legal. We identified 24,267 patients 65 to 67 years old with coronary artery disease in states where marijuana was legal. Among these patients, 7855 had a marijuana term in their notes suggesting that this proposed method of recruitment for a cardiovascular cohort study is feasible. Second, it is unknown whether VA providers are more likely to document marijuana use compared to providers in other health systems. Our cohort construction method should be replicated in other health systems as the availability of a VA search function as well as our ability to centralize all the notes from the sample aided our ability to implement this approach. Third, we had access to contact information and both home and cell phone numbers as part of the electronic medical record. This access significantly aided our recruitment methods. Medical marijuana is also legal in California, which may have also aided the response rate as well as improved the accuracy of documentation. Finally, the age range of this sample was narrow and the sample size small. The findings may not generalize to younger populations that may be more reluctant to share their use with health care providers. This method to identify marijuana users should be tested in larger datasets that are more representative of the population.

Past research in the health effects of marijuana has been limited because developing sufficiently large cohorts with sufficient use to study has been challenging. In this study, we demonstrate the feasibility of developing large prospective cohorts of marijuana users. Such cohorts can be used to answer important questions regarding the health effects of marijuana in the era of legalization. We also demonstrate that methods that combine information available in the free text of the medical record with patient health interviews provide opportunities for a more efficient approach to the development of prospective cohort studies. Future work should replicate our method of cohort construction in other health systems, and for other health factors and outcomes.
